# Addressing practical challenges of LiB cells in their pack applications

**DOI:** 10.1038/s41598-024-60816-x

**Published:** 2024-05-02

**Authors:** Cher Ming Tan, Yan Yang, K. Jithendra Mani Kumar, Debesh Devadutta Mishra, Tsung-Yu Liu

**Affiliations:** 1 Center for Reliability Sciences and Technologies, Chang Gung University, Taoyuan, 333 Taiwan; 2 Department of Electronic Engineering, College of Engineering, Chang Gung University, Taoyuan, 333 Taiwan; 3 Department of Radiation Oncology, Chang Gung Memorial Hospital, Taoyuan, 333 Taiwan; 4 Center for Reliability Engineering, Ming Chi University of Technology, New Taipei City, 243 Taiwan; 5 Grace Connection Microelectronics Limited, Taoyuan, Taiwan

**Keywords:** SoH (state of health) degradation, Electrode coefficient of polarization, Maximum charge storage capacity, On-line SoH determination, SoC (state of charge), DC-IR, Electrical and electronic engineering, Energy storage

## Abstract

In a battery pack, several lithium-ion batteries (LiBs) are connected in series and parallel so that sufficient voltage, current and power can be provided for applications. To ensure safe operation, when one of the LiB cells in a pack has its SoH below 80%, the entire pack will have to be discarded. Thus, ensuring all the LiB cells degrade similarly in a pack is crucial to maximize the potential of all the cells in a pack. There are several methods to perform screening on the LiB cells for such purpose, but there exist many practical challenges for estimating and predicting the degradation rate of the cells before they are chosen to be put in a pack which will be described in this work. This work provides solutions to some of these challenges and shows through experiments that one can screen the weak cells from production batch with just the first discharge cycle, and one can also predict the statistical distribution of the degradation rates of LiB cells in a production batch. On-line in-situ determination of the SoH of each cell connected in a pack is also made possible with a solution presented in this work, and this method is verified over many different types of LiB from various manufacturers.

## Introduction

**Figure 1:**
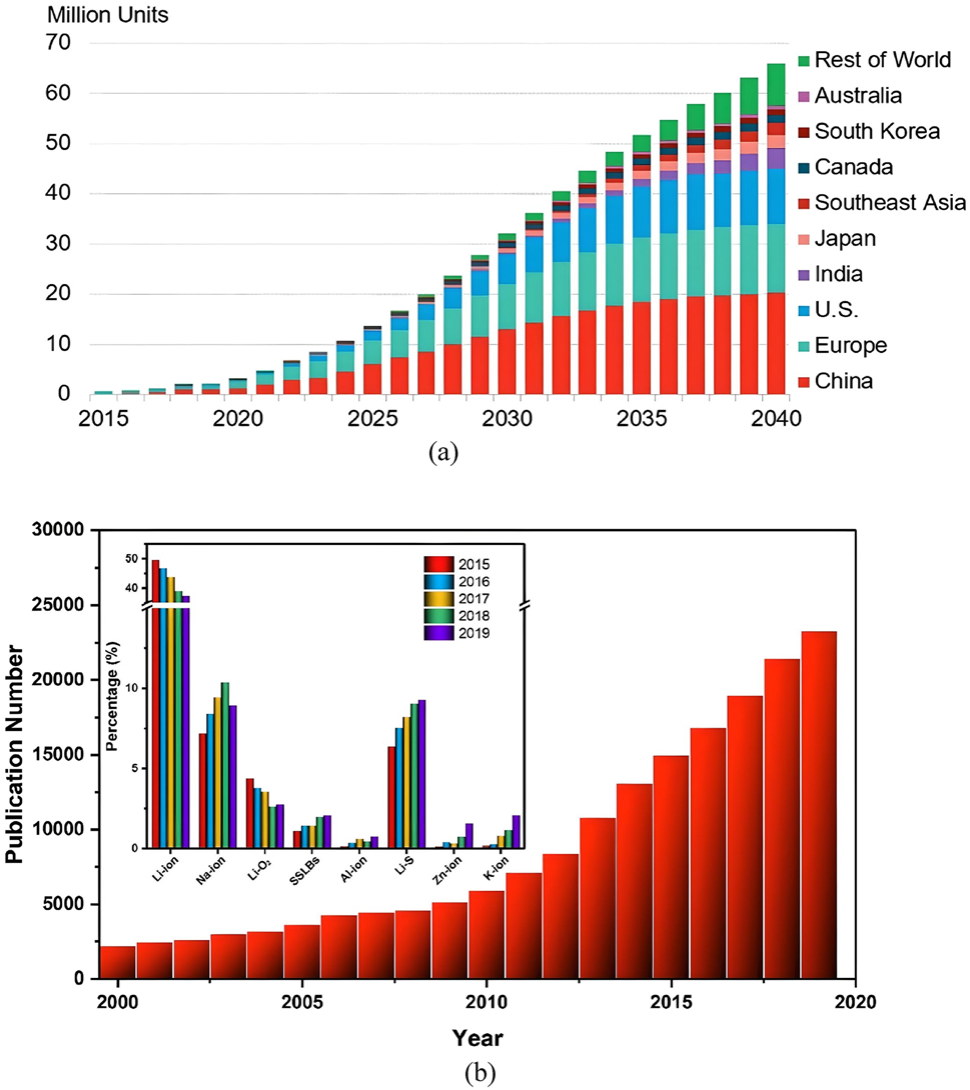
(**a**) Global market growth and projection of LiB^1^ and (**b**) number of publications on Li batteries (the inset displays the percentage of papers published for different types of Li Ion batteries from 2015 to 2019)^2^. Copyright 2021 IOP-Science.

Lithium-ion battery (LiB) is an important technology device today for environmental sustainability. In particular, it is an essential device for electric vehicles and energy storage systems. The market growth and the projection of LiB can be seen in Fig. 1a, and correspondingly, extensive research on the LiB can be seen in Fig. 1b, indicating its importance.

While LiB has been employed widely, and despite the extensive research on it, several challenges remain to be tackled. Generally, LiB is used in a battery pack which consists of many LiB cells connected in series and parallel, for the provision of adequate power and energy. Consequently, a few key practical challenges regarding the quality and reliability of the cells and pack, apart from safety, are present as follows:

1. Estimation of the reliability of each LiB cell before they are welded together to form a pack, particularly the prediction of its rate of degradation in the pack during the usage of the pack.
2. Accurate determination of the charge storage capacity of individual LiB cell in a pack during its operation life cycles, without disconnecting the cells.
3. Accurate determination of deliverable charge of a battery pack at different discharge currents and temperatures during its operation.
4. Estimation of the remaining useful life of the cells and the pack.

Challenge #1 arises due to the requirement that the State of Health (SoH) of each LiB cell in a pack cannot go below 80% for safety consideration in current practice^3^. While the SoH of each cell is 100% at the start of use, if just a LiB cell in a pack degrades too fast due to its poor reliability and/or other factors, the entire pack must be replaced, regardless of the SoH of other cells. Therefore, it is crucial to ensure the reliability or the rate of degradation of each cell in a pack to be as uniform as possible during their operation. This leads to the necessity of a good pack design to ensure nearly equal operating and environmental conditions for each cell, and this is indeed another challenge to consider for a battery pack which is beyond the scope of this work. In fact, even with good pack design that fulfills the above-mentioned criteria, if the cells are of different reliability and hence their degradation rate of SoH are different at a given operating environment, the benefit of good pack design will be voided.

SoH (State of Health) mentioned in the above is a good indicator of the health deterioration of a battery. Electrochemical mechanisms lead to gradual health deterioration of the battery, and capacity fade and internal resistance growth are two consequences of battery aging. State of Health (SoH) is a percentae-based indicator of battery health, typically defined in one of two ways: either by assessing capacity or by evaluating internal resistance. The prevailing definition of SoH leans towards capacity assessment for batteries given below.

1 $$SOH = \frac{Q}{Qrated}$$

where Q is the capacity of the fully charged battery at current state and Q_rated_ is the rated capacity of the unaged battery. This is also the definition of SoH used in this work.

Challenge #2 is related to the accurate determination of the State of Charge (SoC) of a LiB in a pack. SoC is given by the amount of charge remaining in a cell divided by the maximum charge capacity (Q_m_) of the cell at the beginning of the discharge cycle. If a cell discharges too deeply (or called over-discharge), its lifetime will be shorter, or its degradation rate will increase^4^. As Q_m_ degrades with charge–discharge cycle, and if an initial Q_m_ is used throughout its lifetime, the computed SoC of 10% could already be over-discharged in actuality after many cycles of operation, and this renders an increased degradation rate of the cell unintentionally that could make a pack to expire early. In current industry practice, as Q_m_ cannot be accurately measured, Coulomb counting method is used as the estimation of Q_d_, but this will over-estimate the actual SoC, especially when Q_m_ has been greatly reduced.

Challenge #3 is on the determination of the deliverable charges from LiB pack under different operating conditions. It is known that the actual charge deliverable Q_d_ from LiB is always less than the sum of maximum stored charge in each cell in a pack, and the difference varies with the cells, even with the same cell after different charge–discharge cycles. The difference depends on the discharge rate and operating temperature of each cell in a pack; and the discharge rate of each cell in turn depends on its SoH. However, it is the actual charge deliverable that is important to users. The ability to determine these charges enables an accurate estimation of the usage capability, either the distance that can be further traveled for EV (Electric Vehicles), or the amount of energy that can be delivered to grid for energy storage system. In this work, only Challenges #1 and #2 will be discussed, and thus we will not elaborate further on Challenge #3.

The last challenge is important because users need to know beforehand when to replace a pack. Current practice is to assume each pack to last for a pre-determined number of years^5^, and this could be too early and can add unnecessary cost to the users. Also, to maximize the useful life of LiB pack, degraded pack can be used in stationary energy storage system as the requirement of 80% SoH can be relaxed in such application. This is the second life of LiB pack. However, how long can the pack be used requires the knowledge of its remaining useful life and the Q_m_ of each cell, and this also decide the price of the pack for its second usage. This topic has attracted much research, and the practical solutions are yet to come. Again, no further elaboration on this challenge in this work. In fact, both Challenges #3 and #4 will be presented in our next work, and one important factor to address these Challenges is the accurate determination of individual cell Q_m_ (not Q_d_) connected in a pack during the operation of the pack, and this will be addressed in this work.

In this work, we aim to address critical challenges associated with the operation and management of lithium-ion battery (LiB) packs, particularly focusing on the selection of cells and the estimation of the State of Charge (SoC) for each cell within a pack. The first two challenges pertain directly to maintaining the safety and efficiency of LiB cells: ensuring that the SoH of each cell does not fall below 80% to avoid premature pack replacement, and accurately determining the SoC to prevent over-discharge that can accelerate cell degradation. While existing literature provides various methodologies to tackle these issues, they often fall short in practical scenarios. Our goal is to offer robust solutions that not only address these challenges effectively but also keep practical applicability in mind, thereby enhancing the operational reliability and cost-efficiency of LiB packs.

It is also to be noted that the first two challenges are on the individual LiB cell in a pack, namely the selection of cells and the estimation of the SoC of each cell in a pack. Proposed solutions to the first two challenges are available in literature, but they have several limitations for their practical applications as will be discussed later. The objective of this work is to provide practical solutions to the above-mentioned challenges with their practical applications in mind.

## Experimental techniques and analysis

Battery charge and discharge experiments are performed in this work. All the experiments are performed at a constant ambient temperature of 25 °C to minimize the effect of the environment, and the charging/discharging currents are also kept constant using battery tester Neware ABT-E408T-5V300A.

The charging process is conducted at a fixed charging rate of 1/2C in CC mode and a voltage of 3.6 V in CV mode with a charge-termination current of 1/20C, according to the manufacturer specification. All battery cells are discharged to the cut-off voltage of 2.5 V as specified in the manufacturer specification. The terminal voltage and current of each cell are continuously monitored, recorded every 30 s or every 0.01 V change in voltage to obtain sufficient data points.

Q_m_ and Q_d_ are computed from the discharge curve of each cell after each cycle. Q_m_ is computed via the method developed in^6^ which is also called ECBE method, and Q_d_ is computed using Coulomb counting method.

The Coulomb counting method measures the discharging current of a battery and integrates the discharging current over time in order to estimate battery capacity. ECBE is a electrochemistry-based electrical (ECBE) battery capacity calculation We get the battery capacity Qm from this equation:

2 $$\eta \left( t \right) = i\left( t \right)\left( {R_{e} + R_{CT} + k\frac{{Q_{m} }}{{Q_{m} - \mathop \smallint \nolimits_{0}^{t} i\left( \tau \right)d\tau }} + \mathop \sum \limits_{n = 1}^{\infty } \left( {1 - exp\left( { - \frac{t}{{R_{n} C_{w} }}} \right)} \right)} \right)R_{n}$$

LFP (lithium iron phosphates) LiB cells are used in this work as they are more commonly employed. Cells from two manufacturers are purchased for experimentation. The purpose of the selection of the cells is to examine the application of the proposed methods to LiBs from different manufacturers. Obviously, more manufactures and types of LiB will be better to demonstrate the effectiveness of the proposed method, but this will require a lot more resources in both time and cost. It is hoped that our proposed method can trigger further collaboration with LiB manufacturers and users for a wider range of verification.

Table 1 shows the specification of LFP cells obtained from manufacturer A. We select 16 battery cells and run for 500 cycles. Table 2 shows the specification of LFP from another manufacturer B.

**Table 1:** The specification details obtained from manufacturer A.

Battery	Characteristics
Chemical system	LiFePO_4_
Nominal voltage	3.2 V
Capacity	57 Ah typical
Charging condition	CCCV 3.6 V max 1/2C-rate (28.5 A), 1/20C C-rate cut-off
Discharging condition	Constant current, 1/2 C-rate, 2.5 V cut off
Size	302 × 103 × 18.5 mm

**Table 2:** Cell specification and experimental parameters.

Battery	Manufacturer B
Chemical system	LiFePO_4_
Nominal voltage	3.2 V
Capacity	310 Ah, 280 Ah, 202 Ah, 100 Ah
Charging condition	CCCV 3.65 V max 1/2C-rate,1/20C-rate cut-off
Discharging condition	Constant current, 1/2 C-rate, 2.5 V cut off

## Addressing the challenges

### Proposed solutions to Challenge #1

To evaluate the reliability of LiB cell is challenging as its acceleration is limited owing that the temperature of LiB cannot go too high for safety consideration and the possibility of changing chemical kinetics at higher temperature that renders extrapolation invalid. Furthermore, as LiB is an electrochemical system, the reliability of a sample set of LiB cells can be quite different from others, in contrast to electronic devices. Consequently, there is a need to have a method to screen LiB cells so that similar SoH degradation rates can be identified before putting them in the construction of a battery pack. Of course, this requires a good estimation of SoH while LiB cell is cycled through its charge–discharge cycles.

However, if the degradation rate of SoH can only be determined through many cycles of charge–discharge cycles, and since screening is to be performed 100%, this will imply that the LiB cells chosen to construct a LiB pack will not be from fresh cells, and hence mere determination of SoH without prediction capability will not be the solution to this challenge.

Current strategies for estimating the State of Health (SoH) of lithium-ion batteries (LiBs) are diverse, encompassing several distinct methodologies, each suited to different aspects of battery management as described in the following.

Model-Based Methods^7–12^: These methods use mathematical formulations to represent battery behavior accurately. They include Electrochemical Models, which simulate internal chemical processes, and Equivalent Circuit Models (ECM), which use simplified electrical components to reflect battery dynamics.

Kernel-Based Techniques^13–20^: These approaches utilize advanced mathematical transformations to manage non-linear relationships within battery data. Popular techniques such as Support Vector Machines (SVM) and Gaussian Processes (GP) provide robust classifications and predictive models, complete with uncertainty estimates. Kernel Ridge Regression and Relevance Vector Machine are also used for their computational efficiency and model sparsity.

Data-Driven Approaches^21–29^: This category uses statistical models and machine learning algorithms, including neural networks and decision trees, to analyze historical battery data. These models are particularly effective at identifying patterns from charge/discharge cycles, voltage behavior, and temperature variations. Deep learning extends these capabilities further by analyzing complex sequences for dynamic battery management applications.

Dual Self-Attention Multivariate Time Series Estimation Network^30–32^: This innovative network employs deep learning to process multivariate time series data from battery sensors. It features dual self-attention mechanisms that capture both intra-variable temporal dependencies and inter-variable interactions, significantly enhancing predictive accuracy. The integration of attention layers with recurrent or convolutional neural networks helps handle the data's complexity, delivering precise SoH predictions essential for the safety and efficiency of battery-operated systems.

These methodologies are employed to gauge the health of the batteries. Unfortunately, none of them have the prediction capability. Also, they cannot provide the determination of SoH when LiB cells are already connected in a pack. This is necessary as will be elaborated later.

To overcome the problem, we propose to perform three tests concurrently, namely a test to determine the Q_m_ distribution of a batch of LiB cells from its sample set; a test to identify poor LiB cell from a batch that might escape from the sample set; and a test to determine cell SoH continuous for individual cells which are already connected in a pack.

**Figure 2:**
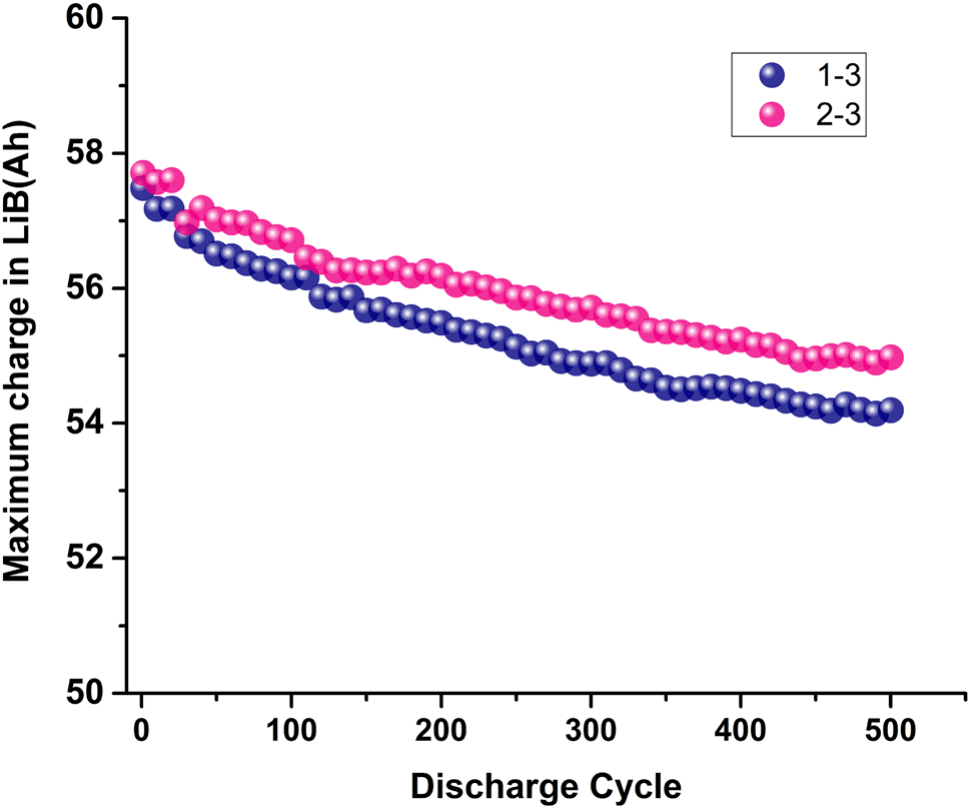
Different degradation rates of LFP LiB with similar Q_m_ at time zero at 1/2C discharge. Cell 1–3 and 2–3 represents two different LiB cells.

The first test is to examine the consistency of LiB cells quality from a production batch. A batch with non-uniform quality is unlikely to have the close degradation rate among them. In fact, even with uniform quality at time zero cannot ensure that they may degrade similarly over time. Figure 2 shows an example of such in our experiments where the quality indexes are Q_m_ or Q_d_. Both cells have the same Q_m_, but their degradation rates are different.

Would there be an appropriate quality index that can predict cell reliability? In fact, reliability generally cannot be predicted from time zero quality as has been demonstrated by one of the authors in^33^. Thus, one should examine the cells’ SoH degradation from a set of sample in a production batch, and apply statistical methods to infer the uniformity of the SoH degradation of all the cells in the batch.

Jeng and Tan^34^ addressed this challenge of assessing LiB reliability with limited degradation data. They introduced an approach that combined a semi-empirical model of LiB with electrochemistry principles to reduce the reliance on extensive degradation data. They highlighted the importance of unit-to-unit variability in LiBs, which hindered the use of basic stochastic models. Instead, they employed a nonlinear random coefficient degradation model to address the nonlinear degradation patterns inherent in LiBs. Their study demonstrated that accurate LiB reliability predictions could be made with less than half of the total life cycle data. They also showed the impact of the variation of LiB cells reliability on pack, and the severity of the impact depends on the pack configurations. Their method also helps to figure out whether the range of lifetimes within a LiB batch was suitable for pack construction, avoiding issues of premature failure. Furthermore, their degradation path model allowed for predicting time to failure distributions in LiB packs for a given LiB SoH degradation rate distribution. In other words, their approach is similar to the reliability test for electronic devices, but taking variability among the devices into consideration, and then providing inference to the entire batch of LiB cells. In this way, one can obtain the SoH degradation rate distribution of a given batch of LiB from a set of samples.

The test mentioned above can be useful if all the cells in the sample set are representative of the cells in the batch. Outliers could happen in the production of LiB cells, and thus one must screen them to remove possible outlier cells in a quick manner as it is to be done 100% of the cells in a production batch. This is the second test developed in this work as will be elaborated in section "Screening method for cells that are outliers from a production batch". As it is to be done 100%, hence this test is worth doing only if the first test results indicate that the quality of the batch is good.

**Figure 3:**
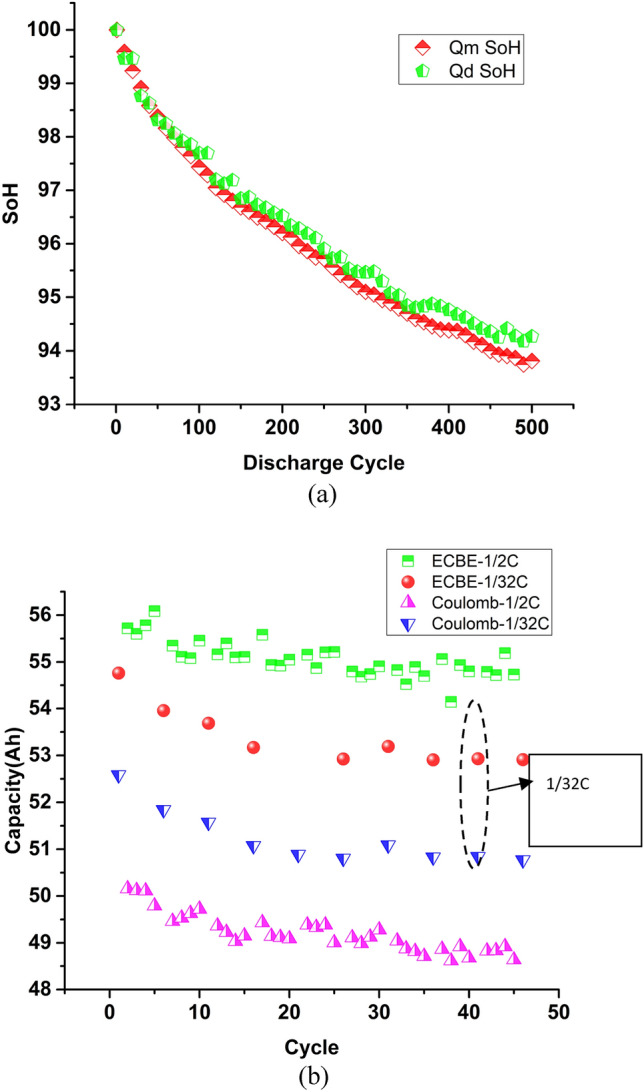
Comparison of (**a**) SoH determination at $\frac{1}{2}$ C discharge and (**b**) LiB capacity using both the Coulomb counting method and ECBE method at $\frac{1}{32}$ C and $\frac{1}{2}$ C respectively. The LiB shown here is 57 Ah LFP.

The third test is needed to verify the effectiveness of the first two tests combined. In the case where some cells do degrade faster than others, despite the above-mentioned two tests, the on-line continuous SoH monitoring can provide information on the weak and strong cells so that pack design can use this information to adjust the operating and environmental conditions of the cells locally to adaptively ensure the cells are degraded uniformly. This on-line SoH monitoring is based on the work by Feng et. al.^6^, and it is called ECBE method. This method derives the Q_m_ value directly from its terminal voltage vs discharge time of a cell during its discharge, and this voltage is the common electrical parameter that will be measured in all battery management systems. Figure 3a shows its comparison with the Coulomb counting method. Figure 3b shows how Q_d_ approaches Q_m_ when the discharge current decreases which also corresponds to lower internal loss.

From Fig. 3a, one can estimate SoH from Q_d_ via Coulomb counting method. However, information shown in Fig. 3b requires the determination of Q_m_, and the difference of Q_m_ and Q_d_ provides crucial information for the screening of poor LiB cell as will be seen later.

The trend shown in Fig. 3a is also observed over more than 20 LiBs tested with capacity ranging from LCO coin cell: NMC 2 Ah (18,650 cells) to LFP 300 Ah cells from different battery manufacturers at different discharge rates^6,35,36^.

Figure 3b depicts Q_d_ approaching Q_m_ as discharge current used in Coulomb counting method decreases. At C/2 discharge rate, the error in cell charge capacity (using Q_m_ as reference) is 11.4%, and at C/32 discharge rate, the error reduces to 4% as expected. The capacity as determined from ECBE is higher for C/2 discharge rate is believed to be due to higher internal heat generated with larger current, and hence Q_m_ is higher^35^.

### Proposed solutions to Challenge #2

The Coulomb counting method is often used to estimate lithium-ion battery state of charge (SoC) and maximum deliverable charge capacity (Q_d_). It works by continually monitoring the current drawing out from a battery and integrating this data over time to compute the total charge delivered. SoC is calculated as a percentage by comparing the remaining charge in a cell to its maximum capacity. The maximum capacity can be obtained by having the discharge that drains all the charges, i.e. SoC = 0% in principle.

However, there are challenges with this method. It relies on precise current integration, and even small errors can accumulate over time, leading to inaccurate SoC estimates. But these can be resolved through good circuit design in BMS (Battery Management System). Battery aging that leads to lower Q_m_ (i.e., the denominator of SoC formulas), calibration requirements, and temperature variations further complicate accuracy^36–38^. Unfortunately, one cannot determine the Q_m_ often as this will shorten the battery life.

A method for the determination of Q_m_ is to use the Open Circuit Voltage (OCV) method. This method is based on the principle of Nernst Equation where the total amount of charge decides the electrode potential at equilibrium. However, practical application of this method presents several hurdles. The dynamic nature of battery aging, which gradually diminishes cell capacity over time, poses a challenge to the OCV method's ability to faithfully represent the battery's actual state. The presence of non-ideal behaviors in real-world batteries, stemming from factors such as high discharge rates and variations in cell chemistry, introduces further complexities, potentially compromising accurate Q_m_ estimation^39–42^. More importantly, the OCV method cannot be used to estimate Q_m_ when a cell is connected with other cells as in a battery pack.

The ECBE method described earlier will be able to address this challenge of Q_m_ determination. However, the computation resources needed were too high and the time for computation was too long for practical applications. Recently, a breakthrough in computation was made and can be found in two patents^43,44^. Using i7 core PC with 32 GB RAM running on window 11, computation time required is only 3 milli-seconds. The accuracy of ECBE has been shown in Fig. 3.

With the in-situ and on-line determination of Q_m_, one can determine the SoC accurately avoiding the problem of un-intentional over-discharge. It also laid the foundation for challenges #3 and #4 which will be discussed in our next work.

## Screening method for cells that are outliers from a production batch

The current common methods of screening of LiB cells before they are put in a pack are the EIS method and Resistance method. In the realm of experimental methods, the internal resistance of a battery assumes a pivotal role in the assessment of its health status. This resistance is indicative of the voltage drop that transpires when a current is applied to the battery. This voltage drop in turn is due to the electrode polarization^45^. As LiB degrades, electrode polarization also increases. If a cell is an outlier with respect to degradation rate (i.e., has larger degradation rate), its electrode or SEI (Solid Electrolyte Interphase) layer will be different from others, and such electrode/electrolyte interface quality can be reflected in the electrode polarization.

A straightforward method to measure resistance is according to Ohm’s Law, i.e., via I-V method, either DC-IV or AC-IV. These have been a common method in industries^46–48^, but they are shown to be inaccurate as discussed in Appendix A (Supplementary materials). The reason of the inaccuracy is because the cell “internal resistance” is due to the electrode polarization that consists of ohmic polarization, concentration polarization and activation polarization, and the latter two do not follow Ohm’s law^49^.

Another technique for determining battery internal resistance is Electrochemical Impedance Spectroscopy (EIS), as illustrated in^50^, which enables the computation of resistance based on the measured impedance. In a noteworthy investigation by^51^, the use of EIS unveiled two prominent aging mechanisms: one linked to charge transfer at the electrodes and the other involving the transport of lithium ions across the Solid Electrolyte Interphase (SEI) layer. EIS can thus reveal potential “internal defects” in a LiB cell, which can presumably reflect its health status and SoH degradation rate. While there are commercial chips now that can perform EIS testing, and hence the cost and time of testing can be low, in-situ and on-line EIS testing of a cell connected with other cells in a pack cannot be done, and this is necessary for challenge #2.

Along with the same argument on “internal defects” finding that could identify outlier cells, ultrasonic inspection is another alternative. It can detect internal aging-related changes within batteries using a wave-generating test bench^52^, but it is time consuming and costly, although recent development has shown significant progress.

Ultrasonic inspection entails the transmission of ultrasonic waves through an object under examination, enabling the meticulous and real-time assessment of various factors, including material properties, internal damage, and structural integrity. This versatility places ultrasonic inspection as a promising tool for investigating changes in the fundamental material properties within batteries. When employing ultrasonic methods to screen battery cells, Time of Flight (TOF) measurements often play a crucial role in the testing procedure^53^.

In the context of battery cell screening, ultrasonic waves are directed into the cell, and the elapsed time for these waves to return offers valuable insights into the cell's internal structure and integrity. However, it is important to note that comprehensive screening process for battery cells encompasses several stages, including setup, calibration, data acquisition, and subsequent analysis. The cumulation of these steps collectively determines the duration required for the screening process, and it may take several minutes to hours.

Ultrasonic testing, despite its many advantages, does come with its share of drawbacks. One challenge is the difficulty in inspecting materials that are small, rough, extremely thin, or have irregular shapes. Additionally, this method demands accessibility to the surface being tested, and it may miss defects close to the surface, particularly in thin steel plates due to the presence of a dead zone^54^. Moreover, ultrasonic testing can be less effective in detecting linear faults aligned parallel to the direction of the sound beam, necessitating a higher level of training and expertise compared to some other non-destructive techniques. To ensure accurate results, reference standards are essential for equipment calibration and defect characterization. Lastly, it is worth noting that ultrasonic testing tends to be relatively costly than alternative inspection methods^55^.

For individual cell screening, the EIS method is possible. However, there is one key parameter, electrode polarization coefficient, that EIS cannot provide, and this parameter can be a direct quality index to the electrode/electrolyte interface integrity as will be elaborated later. While Ultrasonic method has been demonstrated for cell production, it is still expensive and only for manufacturers.

**Figure 4:**
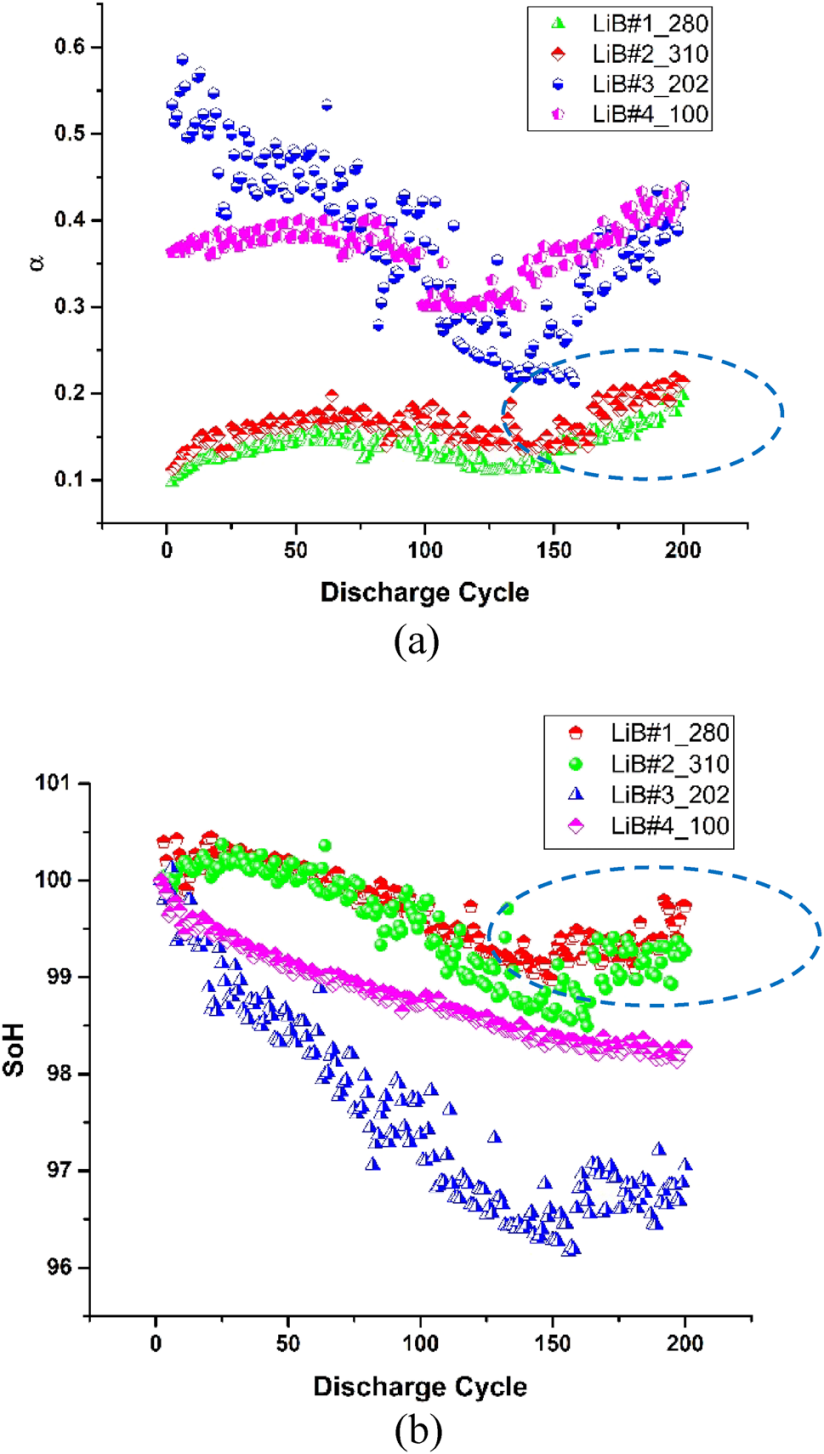
(**a**) Variation of α with discharge cycles for different batteries; (**b**) SoH degradation of different batteries.

To provide a quick and cost-effective solution for screening, we propose to use electrode polarization coefficient. Section "Electrode polarization coefficient" and Appendix B (Supplementary Materials) provide the detail of this coefficient. Figure 4 shows our experimental results that demonstrate the application of this electrode polarization coefficient (α) to identify outlier cells. These cells are LFP LiB with different capacities, and they are from manufacturer B. In comparison of Fig. 4a,b, we can see the following:

- LiB with higher Ah rate will have lower α values. This is expected as cell with higher Ah rate will have a large electrode area, and α is the coefficient of polarization per unit area (see Appendix B (Supplementary Materials)).
- α values grow initially that represents the growth of SEI layer (see Appendix B (Supplementary Materials)).
- LiB#3 has abnormally high initial α value, and its value decreases instead of increases at the beginning. This indicates poor electrode/electrolyte interface, and hence its SoH degradation rate is also the worst.

This electrode coefficient of polarization was first introduced by Shepherd^56^. However, due to its difficulty in obtaining its value, this parameter was usually omitted. As we have modified the ECBE model such that we can now perform calculations in a short time, both Q_m_ and α values can be obtained simultaneously of individual cell regardless of they are standalone or connected in a pack. In fact, the first 2 cycles can already allow us to identify the poor cells LiB#3 through α.

As this parameter is seldom mentioned, detail description of this electrode polarization coefficient will be presented in Appendix B (Supplementary Materials), and its brief introduction is shown in the next section.

The use of α for screening out weak cells as mentioned above requires only a few cycles to track its trend. From an economical point of view, however, it will be good to remove or identify a poor cell more quickly so that no further measurement is needed on these cells which will be useless but consume resources.

For cells with poor reliability that will degrade rapidly, there can be several mechanisms. A high electrode resistance or a high electrolyte resistance or a poor separator could lead to poor cell, and they can be easily identified through EIS on the fresh cells from the value of R_e_ and the slope of the Warburg element in the Nyquist plot from EIS. In the fresh cell, SEI is in the forming stage, and R_ct_ in the plot could produce non-conclusive results.

To reveal the defect at the electrode/electrolyte interface of a fresh cell which is usually the main cause of poor reliability for a cell, a different method is required. Such defect can be surfaced up if the internal loss due to the interface defect can be monitored when a current is forced through it via discharge. This can be done by comparison the Q_m_ obtained using ECBE and the Q_d_ obtained using Coulomb counting. Their difference (called ΔQ) can be easily obtained as each fresh cell will go through a complete discharge and this discharge curve (at the first cycle) will allow us to obtain both the Q_m_ (through ECBE method) and Q_d_ (through Coulomb counting method).

For the cells shown in Fig. 4b, LiB#3 has poor reliability. Table 3 shows the comparison of ΔQ for all the cells shown in Fig. 4, and one can easily see that ΔQ is the largest for LiB#3 (as indicated by an arrow).

**Table 3:**
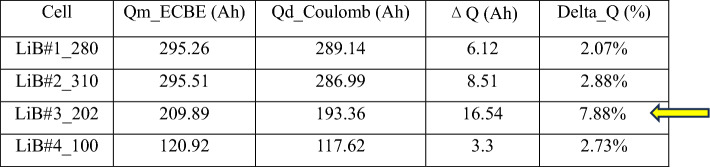
Comparison of ΔQ for the cells in Fig. 4b.

**Figure 5:**
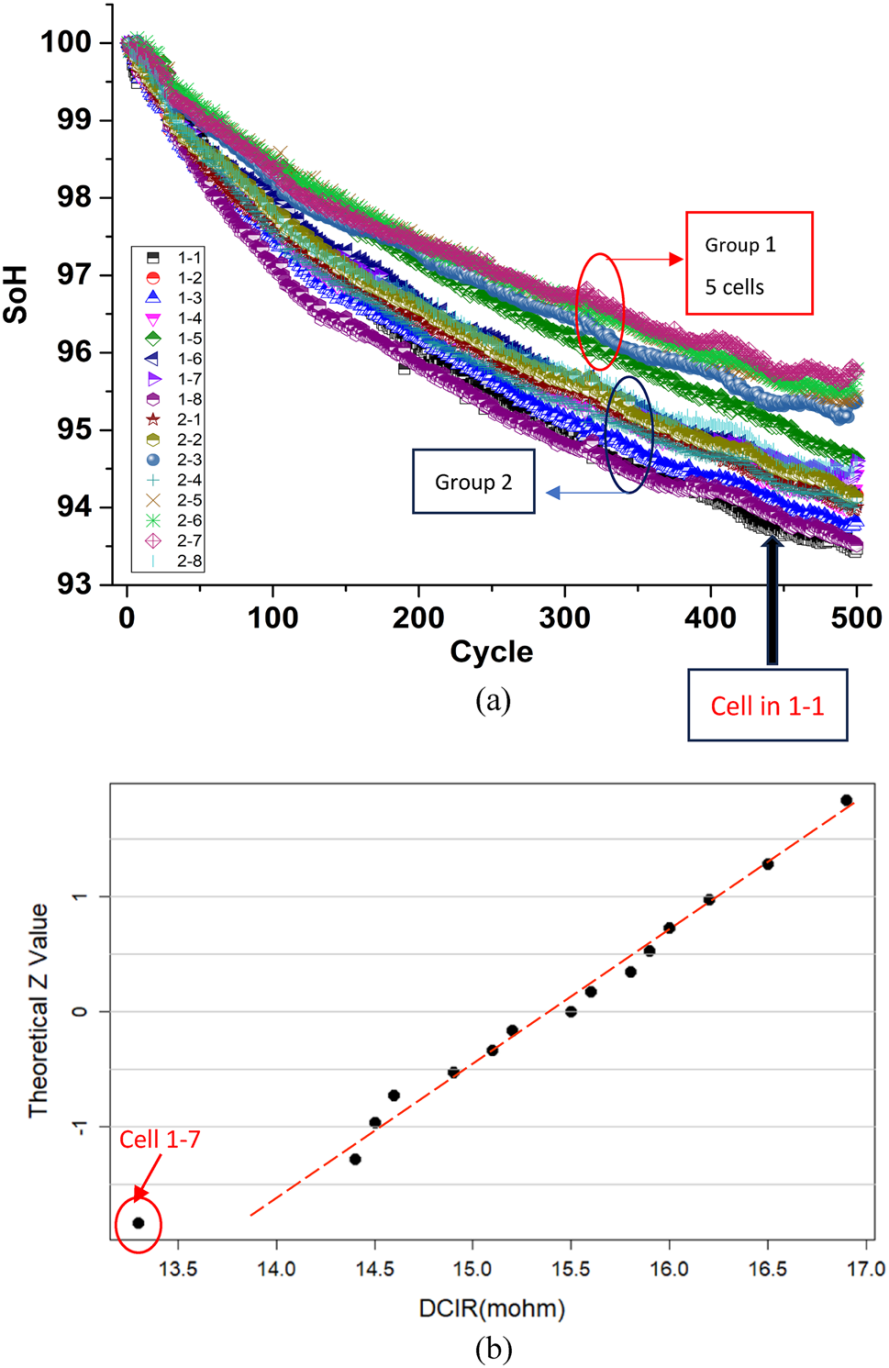
(**a**) SoH degradation with cycles of 16 LFP LiB cells, showing at least two distinct groups. The labels in legends represent the channel number of our battery tester; (**b**) Q–Q plot of DC-IR of the same 16 cells, showing no indication of two groups.

We also performed a similar test on 16 LFP cells of around 60 Ah from manufacturer A. Figure 5 shows the SoH plot of the 16 cells, and we can see that there are 2 groups of cells among the 16 cells. However, DC-IR method cannot differentiate between them as can be seen in the normal probability plot of the IR values obtained (Fig. 5b).

**Figure 6:**
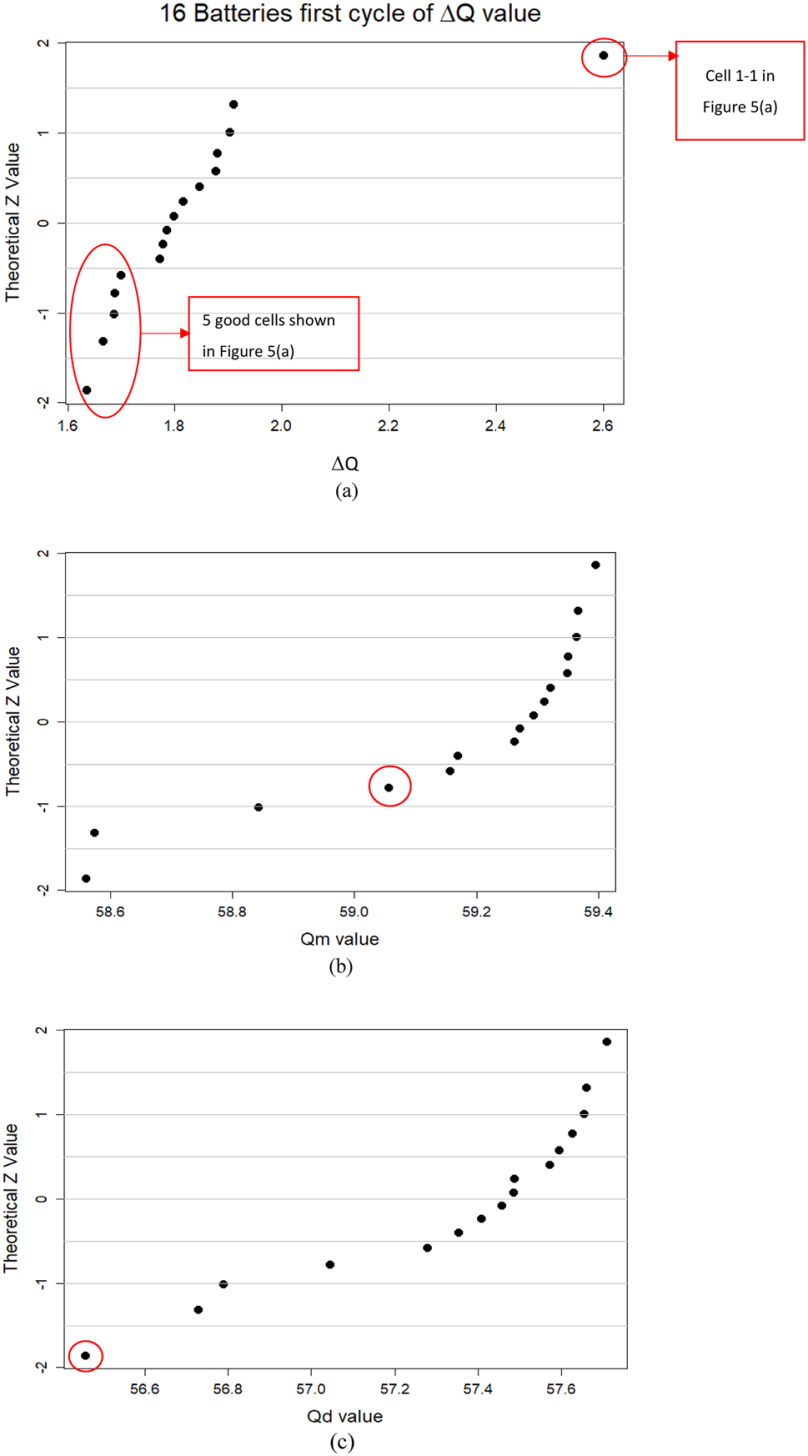
(**a**) Q–Q plot of ΔQ showing the ability to differentiate between reliable and poor cells from just the first cycle of charge-discharging; (**b**) Q–Q plot of Q_m_ which does not show the ability of differentiation. The point with red circle is the poor cell 1-1. (**c**) Q–Q plot of Q_d_ which does not show the ability of differentiation. The point with red circle is the poor cell 1-1.

By using the ΔQ method, the two groups can be seen clearly as shown in Fig. 6. In fact, one of them, cell 1-1 is especially weak, and this is also shown in the SoH plot in Fig. 5a. Also, the good cells shown in Fig. 5 can also be grouped correctly using our ΔQ method, simply from the first cycle of discharge data. Interestingly, by using merely the Q_m_ or Q_d_ values, such differentiation cannot be made as shown in Fig. 6.

## Electrode polarization coefficient

**Figure 7:**
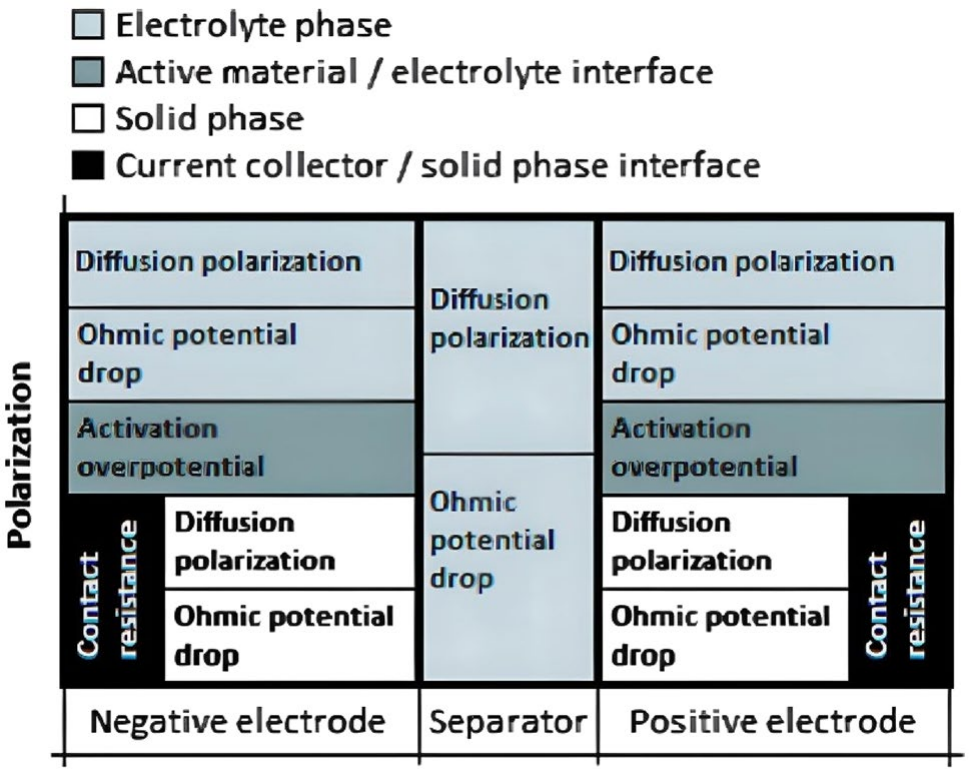
The sub process related polarizations within a lithium-ion battery cell. Copyright 2010 IOP-Science^57^.

The overpotential observed in the discharge curve is due to the polarization in the electrochemical cell. Figure 7 shows the different types of polarization observed^57^. The polarization consists of three groups of subprocesses as follows:^58^

1. Activation of the electrochemical reactions.
2. Mass transport of species.
3. Inadequate contact between different phases and materials in the electrodes.

The driving force of polarization is the current passing through the cell, which causes a deviation of the electrode potential or the cell potential difference from their equilibrium value^59,60^.

The part of the polarization that is associated with the activation of the electrochemical reactions is called the activation overpotential in this study. Polarization due to mass transport can be due to the concentration gradients that are built up in the electrolyte and in the solid phase, which is called the diffusion polarization; the insufficient ionic conductivity in the electrolyte (due to separator integrity and Warburg element) and the insufficient electronic conductivity in the solid phase are the contributors to the ohmic potential drops.

The third group of subprocess occurs both between the current collector and the porous matrix, and between the active and conductive materials in the positive electrode. The polarization due to the former effect is denoted as contact resistance.

Electrode Polarization coefficient, often denoted as α or K, is a parameter used to characterize the activation polarization behavior of an electrode in an electrochemical cell or battery. Hence it is closely related to the Butler-Volmer equation. It is expressed per unit active material current density, and it can be mathematically represented as follows^56,61^:

3 $${{\varvec{\upalpha}}} = {\mathbf{dE}}/{\mathbf{di}}$$

where dE is the change in electrode potential (overpotential) and di is the change in the current density passing through the electrode.

This parameter is essential in understanding the performance and efficiency of electrodes in electrochemical devices, such as batteries, fuel cells, and electrolyzes. It gives insight into the kinetics of the electrode reactions and how the current density may affect the potential behavior of the electrode. As the operation of LiB depends critically on this kinetics, this coefficient can be a direct quality index to LiB cell, and we have indeed seen from the experimental results presented earlier in Fig. 4. The electrode coefficient of polarization is primarily associated with the overpotential related to charge transfer kinetics, and ohmic resistance is typically considered separately.

Appendix B (Supplementary Materials) shows our effort to summarize the potential applications of the electrode coefficient of polarization (α) as deducted from the work of Shepard and others, and it can be seen that α can provide the following information about a LiB cell:

A fast kinetics has low value of α. Thus, the lower the value of α, the healthier is the cell.
2. The charge transfer kinetics is closely related to the SEI layer thickness and its quality, thus the value of α can provide the following information:
  - The rate of increase in the value of α from fresh cell indicates the growth rate of SEI layer during the initial cycles of charging and discharging.
  - An unstable value of α indicates unstable or uneven SEI layer.
  - An increase value of α indicates a reduction of active electrode surface area due to the occupancy of SEI layer, or the reduction of lithium-ion mobility due to degraded SEI layer.
  - Temperature dependence of α value can also shed light on the phase transition of electrode materials or change in the solubility of the electrode materials; change in the electrolyte properties, and mechanical stress in electrodes due to thermal expansion.

In view of its importance, determining the value of the coefficient, especially in-situ, will be useful in LiB cells and pack management. The method of determination as presented by Sheperd can be found in^56^, and it requires the cell to be discharged at two different discharge currents or 3 points on a discharge curve. We found that the selection of different points in the two discharge curves can lead to different value of the α as will be elaborated later. His method is also not applicable for LiB cell connected in a pack.

Determination of the Electrode Coefficient of Polarization for practical challenges presented in this work.

To obtain this coefficient, let us go back to the ECBE model^6^. In the model, the terminal voltage across a LiB cell is described by.

4 $$\eta = i\left( t \right)\left[ {R_{e} + R_{CT} + \alpha \frac{{Q_{m} }}{{Q_{m} - \mathop \smallint \nolimits_{0}^{t} i\left( \tau \right)d\tau }} + \mathop \sum \limits_{n = 1}^{\infty } \left( {1 - e^{{\frac{ - t}{{R_{n} C_{W} }}}} } \right)R_{n} } \right]$$

where,

5 $$R_{n} = \frac{{8k_{1} }}{{\left( {2n - 1} \right)^{2} \pi^{2} }}$$

6 $$C_{W} = \frac{{k_{1} }}{{2k_{2}^{2} }}$$

and the rest of the parameters have their usual meaning.

By fitting Eq. (4) to a discharge curve, all the parameters in the equation can be determined, but the time taken will be too long for practical applications. However, if we focus on getting only Q_m_ and α, the computation time becomes in milli-second scale, and the computation algorithm is now hardwired into an integrated circuit produced by Grace Connection^62^.

**Figure 8:**
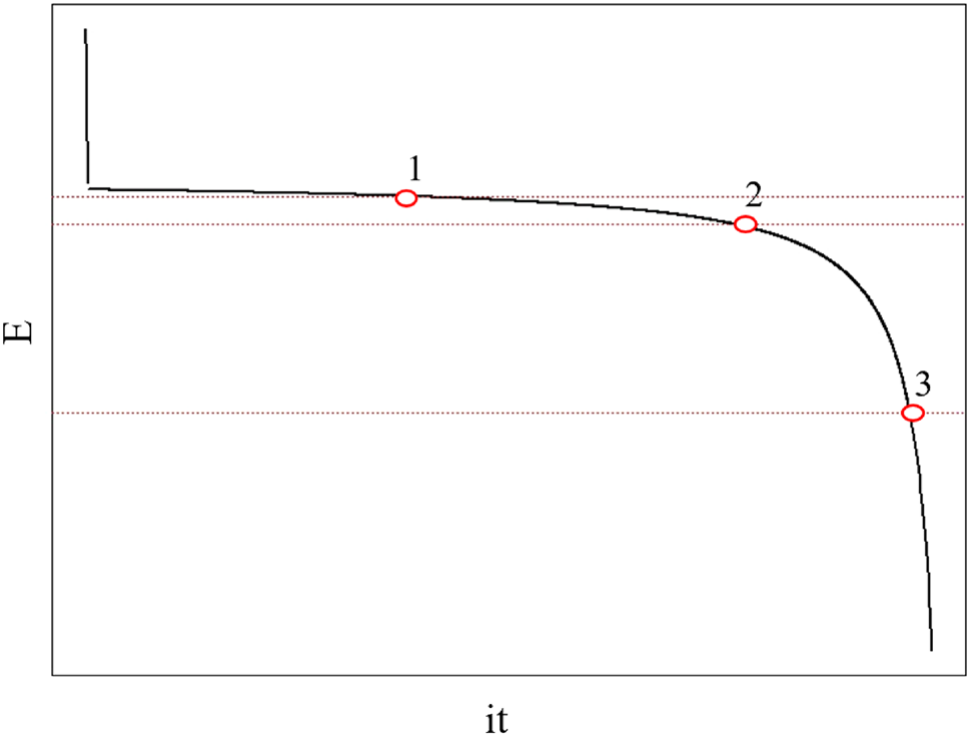
Selection of three points on a discharge curve to compute the value of α as extracted from^56^. The diagram is re-drawn from^56^, and y-axis is the battery terminal voltage, and x-axis is the discharge current multiple the discharge time, i.e. the charge delivered.

Shepherd^56^ provided his method to compute the value of α (or K in his equation). Three points on a discharge curve are needed for the computation as shown in Fig. 8 which is extracted from his paper. In the battery discharge curve, the relationship between voltage and time is given as follows^56^

7 $$E = E_{s} - K\left( {\frac{Q}{Q - it}} \right)i - Li + Ae^{{{\raise0.7ex\hbox{${ - Bit}$} \!\mathord{\left/ {\vphantom {{ - Bit} Q}}\right.\kern-0pt} \!\lower0.7ex\hbox{$Q$}}}}$$

Let us take three points $\left( {it_{1} , E_{1} } \right), \left( {it_{2} , E_{2} } \right)$, and $\left( {it_{3} , E_{3} } \right)$ on a discharge curve, we have the following:

8 $$E_{1} = E_{s} - K\left( {\frac{Q}{{Q - it_{1} }}} \right)i - Li + Ae^{{{\raise0.7ex\hbox{${ - Bit_{1} }$} \!\mathord{\left/ {\vphantom {{ - Bit_{1} } Q}}\right.\kern-0pt} \!\lower0.7ex\hbox{$Q$}}}}$$

9 $$E_{2} = E_{s} - K\left( {\frac{Q}{{Q - it_{2} }}} \right)i - Li + Ae^{{{\raise0.7ex\hbox{${ - Bit_{2} }$} \!\mathord{\left/ {\vphantom {{ - Bit_{2} } Q}}\right.\kern-0pt} \!\lower0.7ex\hbox{$Q$}}}}$$

10 $$E_{3} = E_{s} - K\left( {\frac{Q}{{Q - it_{3} }}} \right)i - Li + Ae^{{{\raise0.7ex\hbox{${ - Bit_{3} }$} \!\mathord{\left/ {\vphantom {{ - Bit_{3} } Q}}\right.\kern-0pt} \!\lower0.7ex\hbox{$Q$}}}}$$

In nearly all cases, the numerical value of $$Ae^{{{\raise0.7ex\hbox{${ - Bit}$} \!\mathord{\left/ {\vphantom {{ - Bit} Q}}\right.\kern-0pt} \!\lower0.7ex\hbox{$Q$}}}}$$ decreases very rapidly, hence its value at points 1, 2, and 3 becomes extremely small and the following can be obtained.

11 $$E_{1} - E_{2} = Ki\frac{{Q\left( {it_{2} - it_{1} } \right)}}{{\left( {Q - it_{2} } \right)\left( {Q - it_{1} } \right)}}$$

12 $$E_{2} - E_{3} = Ki\frac{{Q\left( {it_{3} - it_{2} } \right)}}{{\left( {Q - it_{3} } \right)\left( {Q - it_{2} } \right)}}$$

Taking the ratio of Eqs. (11) and (12), we have

13 $$\frac{{E_{1} - E_{2} }}{{E_{2} - E_{3} }} = \frac{{\left( {it_{2} - it_{1} } \right)\left( {Q - it_{3} } \right)}}{{\left( {it_{3} - it_{2} } \right)\left( {Q - it_{1} } \right)}}$$

Since $\left( {it_{1} , E_{1} } \right), \left( {it_{2} , E_{2} } \right)$, and $\left( {it_{3} , E_{3} } \right)$ are known, Eq. (13) enables us to find Q. Substitute Q into either Eq. (11) or (12), K can be determined.

Using this method, we compute the values of K for different set of the three points, and the results are shown in Table 4.

**Table 4:** Computation and comparison of α values using Shepherd method and ECBE.

S/No	Cell voltage			α(S)	α(ECBE)
	Point 1	Point 2	Point 3		
1	3.284	3.244	2.898	1.21	0.24
2	3.261	3.244	3.022	0.70	0.24
3	3.261	3.244	2.898	0.63	0.24
4	3.261	3.236	3.022	0.48	0.24
5	3.261	3.236	2.898	0.45	0.24
6	3.261	3.217	3.022	0.46	0.24
7	3.261	3.217	2.898	0.43	0.24
8	3.261	3.187	3.143	0.71	0.24
9	3.247	3.236	3.134	0.32	0.24
10	3.247	3.236	3.113	0.32	0.24
11	3.247	3.236	3.022	0.29	0.24
12	3.247	3.236	2.898	0.28	0.24
13	3.247	3.217	3.143	0.43	0.24
14	3.247	3.217	3.134	0.47	0.24
15	3.247	3.217	3.113	0.44	0.24
16	3.247	3.217	3.022	0.36	0.24

From Table 4, we can see that the value obtained depends very much on the choice of the three points. On the other hand, our method, which is determined by fitting Eq. (4) into a discharge curve, does not depend on the choice of the three points, and we give consistent results to the value of α.

## Conclusion

As LiB cells are generally connected into a pack for their applications, there remain several practical challenges with respect to the reliability of battery pack. In this work, we addressed two of these challenges, namely the estimation of LiB cell reliability and the prediction of its rate of degradation during usage while in a pack; and the accurate determination of the charge storage capacity of the cells while they are in a pack during pack operation. Various reported methods were also presented with their limitations that could hinder their practical applications.

Practical solutions for the above-mentioned challenges are developed in this work where 100% fast screening, determination of the maximum charge capacity distribution of a LiB production batch and continuous determination of the cells’ SoH while they are connected in a pack can be done. The solutions are demonstrated experimentally with promising results. An often-omitted parameter in a battery cell, electrode coefficient of polarization is re-introduced and used as an effective quality index for LiB cells, and its effectiveness is verified experimentally. Another factor, ΔQ, which is the difference of the maximum charge capacity, and the maximum deliverable charge of a cell is also introduced, and our experiment shows that it can be used to screen away poor cells effectively with just the first cycle.

Although the experiments presented in this work are with limited number of LiB cells, the promising results could shed light to the solutions of the several practical challenges associated with the use of LiB cells in pack applications. It is hoped that this work can open up further collaboration with LiB manufacturers and users, so that some practical challenges can be resolved, and push the applications of LiB further.

### Supplementary Information

Supplementary Information 1.

Supplementary Information 2.

## Data Availability

The datasets used and/or analyzed during the current study available as “Raw data” uploaded in the “Supplementary Materials” subsection.
