# Supplementary material for: Addressing practical challenges of LiB cells in their pack applications
Source: Sci Rep. 2024 May 2;14:10126. doi: 10.1038/s41598-024-60816-x (PMC11066021; doi:10.1038/s41598-024-60816-x)
Supplement: Supplementary file 2 — Supplementary Information 2. [file 41598_2024_60816_MOESM2_ESM.docx]

**Supplementary Materials**

**Appendix A Issue with DC-IR for Screening LiB-**

A common DC-IR method is performed via the set-up as shown in Figure A.1


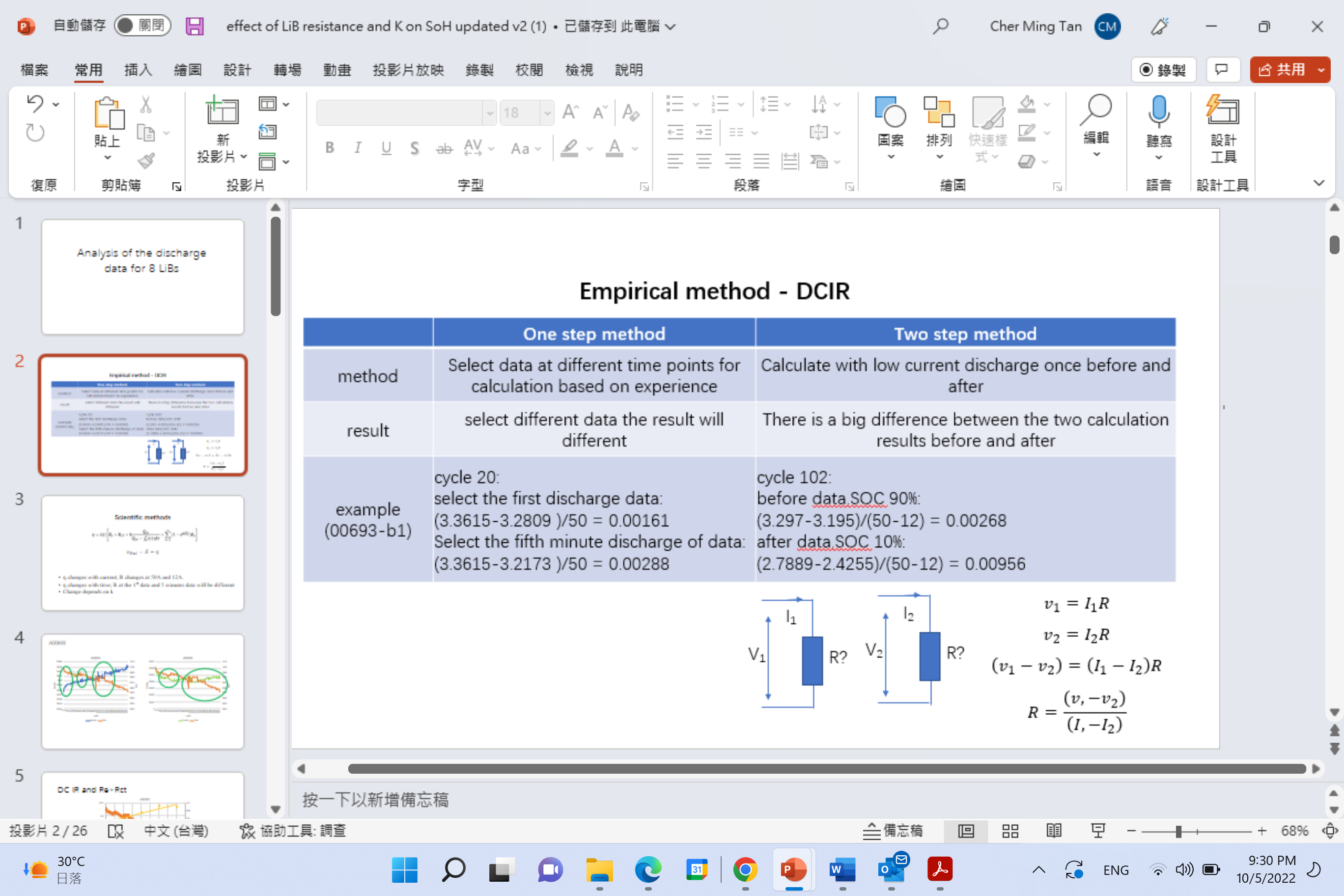


Figure A.1 Schematic of DC-IR set up

Based on Figure A.1, there are two ways to determine the internal resistance of LiB as follows:

$R=\frac{v_{1}}{I_{1}}$ (A.1)

and

$R=\frac{\left( v_{1}-v_{2} \right)}{\left( I_{1}-I_{2} \right)}$ (A.2)

It is found that the time for discharge, discharge current and SoC at the time of measurement will all affect the results of resistance. This is expected as the terminal voltage is depends on the over-potential of the cell, which in turn vary with the discharge current, its SoC and temperature. Most often, the measurement is performed using discharge current pulsing to avoid heating effect. Table A1 shows the difference resistance obtained using current with different pulse width, and one can see a significant different in the resistance obtained, based on Eqn (A.1) [1].

Table A1 Internal resistance of the NX2P0M cell, measured according to Eqn (A.1), as extracted from Reference [1]


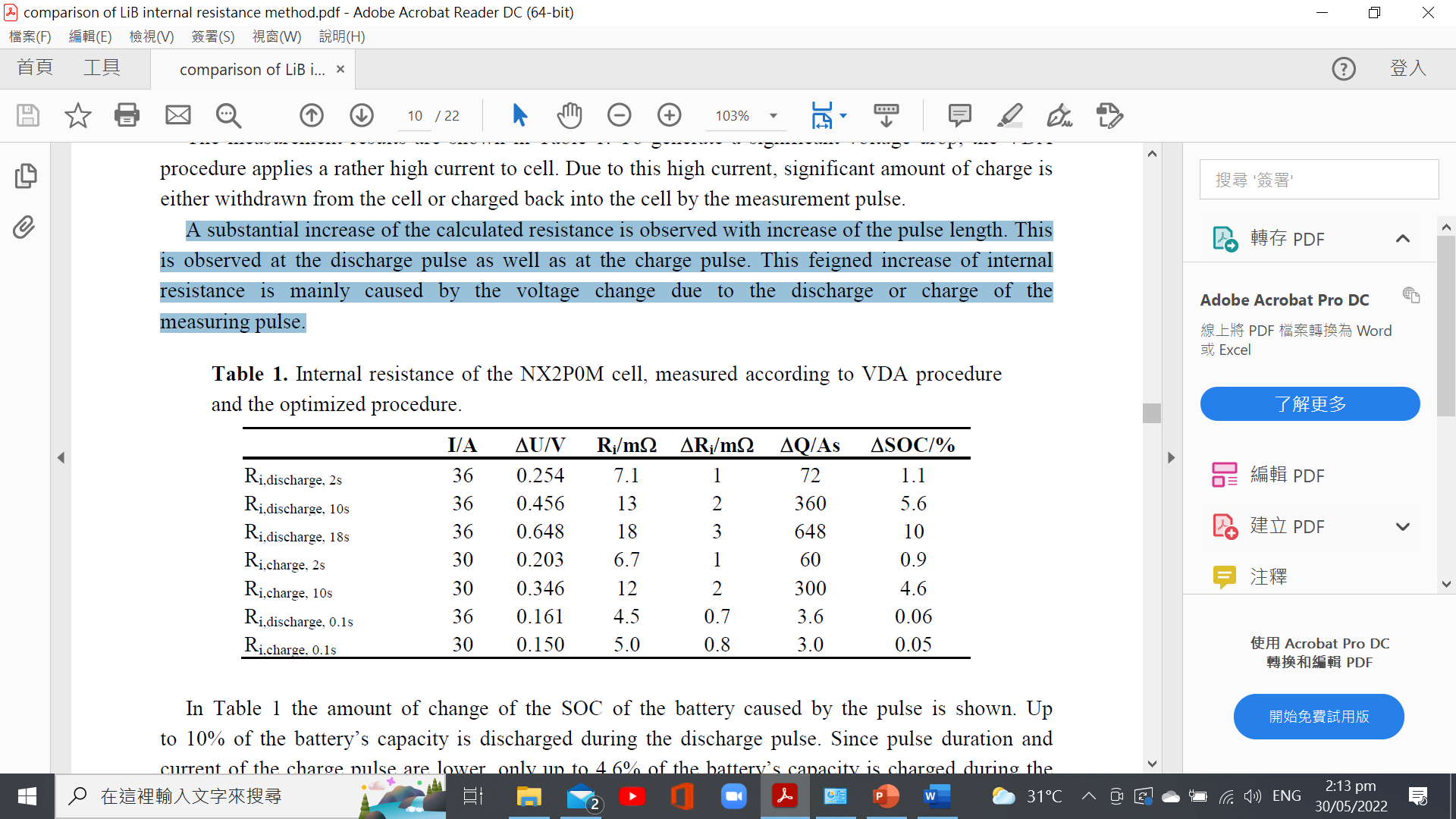


We also performed DC-IR measurement experiments using Eqn (A.2) with two test conditions as shown in Table A2. The LiB is LFP with 60Ah capacity from manufacturer B, and a total of 16 LiB cells were tested.

Table A2 DC-IR measurement set up parameters

| Method | Test set-up parameters |
| --- | --- |
| Method 1 | 12A discharge current for 30 s, then 50A for 30 s. |
| Method 2 | 5.7A discharge current for 10 s，then 57A for 1 s. |

The resistance results obtained at 98% SoC were plotted using Probability plot to examine the distribution of the resistance of the sample set. Figure A.2 shows the plot using method 1 and Figure A.3 shows the plot using method 2.


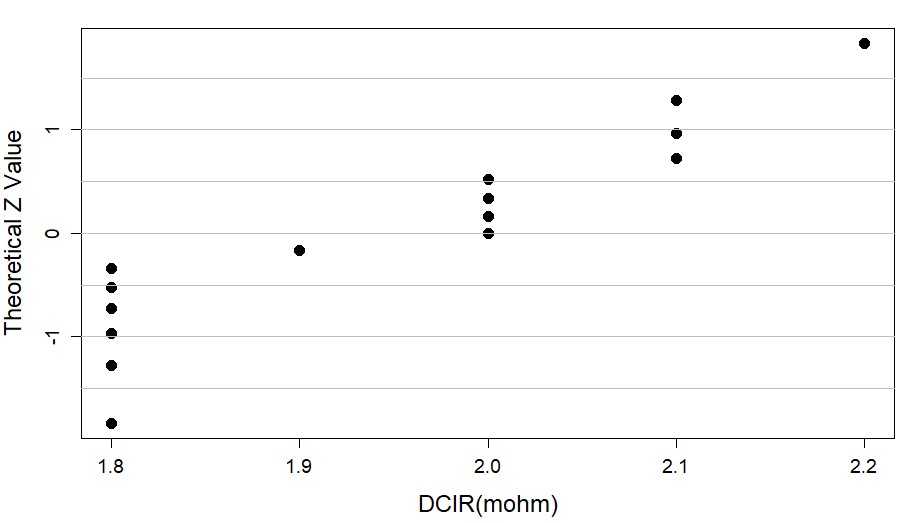


Figure A.2 Probability Plot of cell internal resistance obtained using method 1


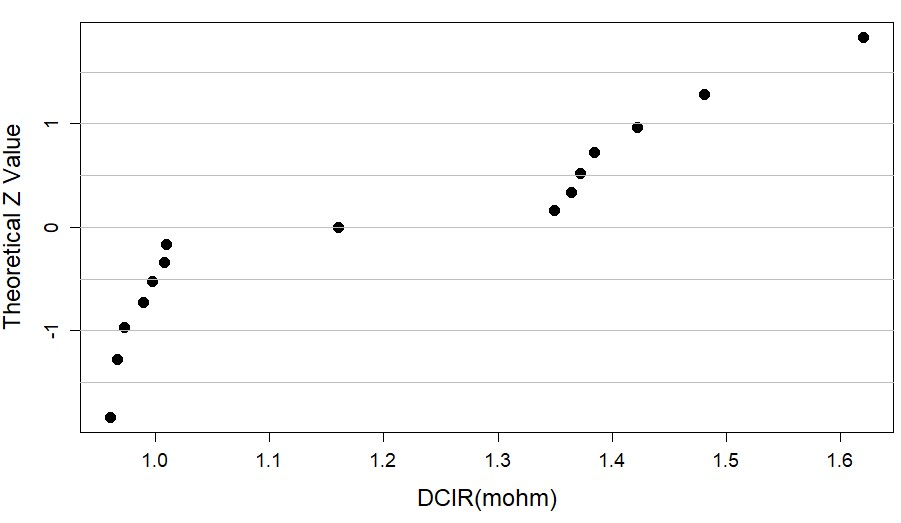


5 good cells

Figure A.3 Probability Plot of cell internal resistance obtained using method 2

The distributions of the internal resistance for the same set of samples were completely different. With method 1, only one distribution can be concluded. However, with method 2, there are two distributions and three outlier points in the sample set, indicating that the sample set does not contain homogeneous cells.

When these 16 LiB cells were subjected to charge-discharge cycle, the degradation of SoH of these 16 cells is shown in Figure A.4


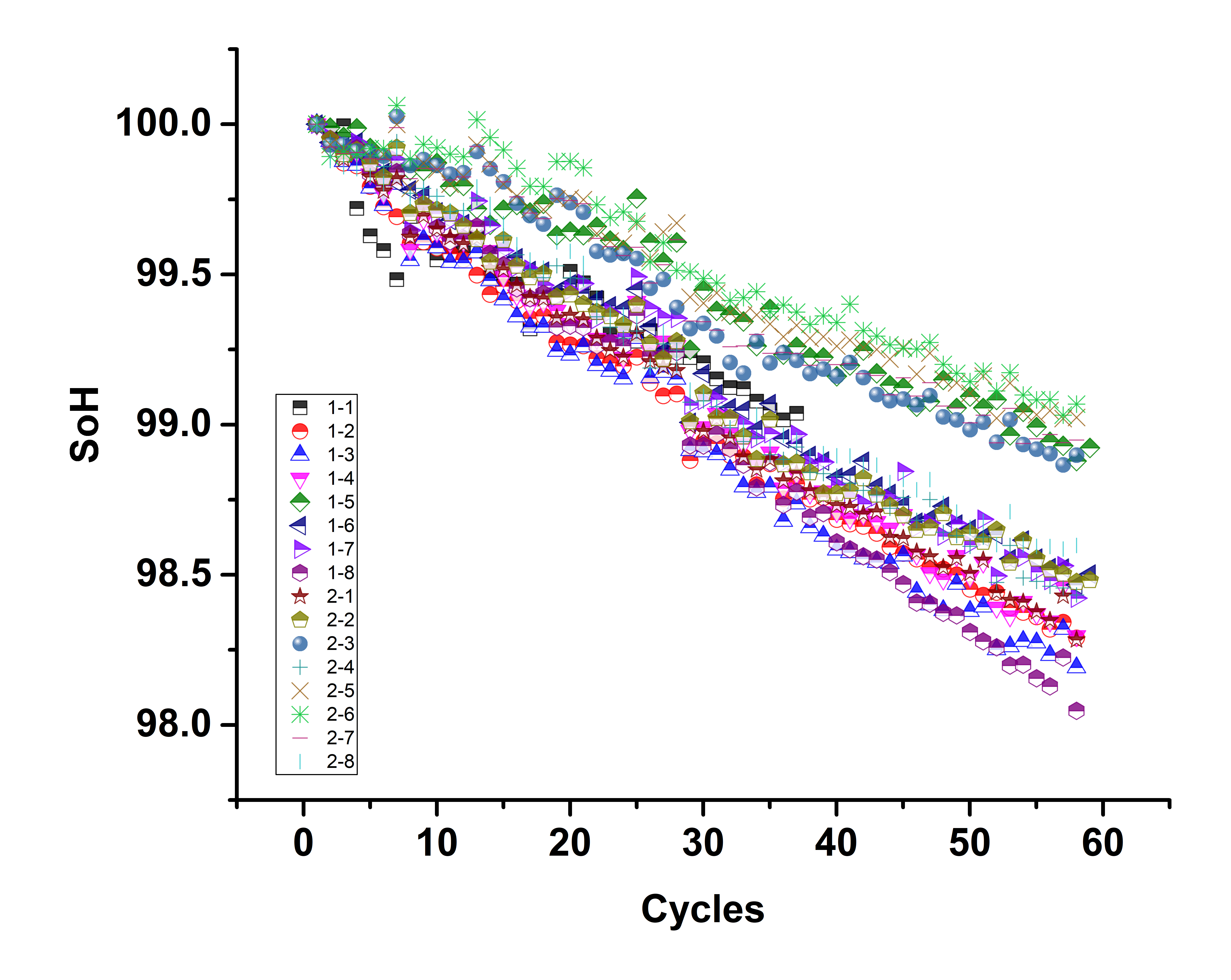


5 good cells

Figure A.4 SoH Degradation of the 16 cells over the charge-discharge cycle. The legend indicates the test channel for each cell.

It is clear from Figure A.4 that the sample set indeed has two groups as revealed by method 2. However, the 5 cells with smaller mean resistance (i.e. the left distribution in the probability plot of Figure A.3) do not correspond to the good 5 cells shown in Figure A.4.

In short, our experiments basically showed that DC-IR test set up parameters can affect the screening result, and even if multiple groups may exist in a batch, DC-IR cannot screen them correctly. Therefore, using DC-IR method for screening can be risky.

Reference

1. Schweiger HG, Obeidi O, Komesker O, Raschke A, Schiemann M, Zehner C, Gehnen M, Keller M, Birke P., “Comparison of Several Methods for Determining the Internal Resistance of Lithium Ion Cells” Sensors, 2010; 10:5604-25. <https://doi.org/10.3390/s100605604>

**Appendix B Literature survey on electrode coefficient of Polarization in Battery**

The concept of electrode coefficient of Polarization (α) was introduced by Shepard [1]. He proposed that cathode potential during discharge is defined by

$$E_{c}=E_{sc}-\alpha_{c}i_{am}$$

where *E_sc_* is a constant potential, α_c_ is the cathode coefficient of polarization per unit active material current density and *i_am_* is the active material current density. The underlying physical mechanisms of the electrode coefficient of polarization (α) can be understood through the processes occurring at the electrode-electrolyte interface in an electrochemical system.

It is important to note that the electrode coefficient of polarization, α, is a phenomenological parameter that is often determined empirically through experimental measurements. It does not explicitly capture all the complex physical chemistry processes occurring at the electrode-electrolyte interface. However, it serves as a useful tool in characterizing and understanding the behavior of electrochemical systems, allowing engineers and researchers to optimize and design efficient electrochemical devices.

Based on the concept of the electrode coefficient of polarization, the SEI (Solid-Electrolyte Interphase) layer can significantly affect the electrode coefficient of polarization (α) and the overall kinetics of charge transfer reactions in an electrochemical system. The SEI layer is an important component that forms on the surface of the electrode, particularly in lithium-ion batteries and other lithium-based electrochemical systems, during the initial cycles of charge and discharge. With the understanding of SEI and α, the expected impacts of SEI layer on α are as follows:

1. Increase in Charge Transfer Resistance: The SEI layer is an electronically insulating layer that hinders direct contact between the electrode material and the electrolyte. As the SEI layer forms and thickens, it increases the charge transfer resistance at the electrode-electrolyte interface [2]. This additional resistance can lead to higher overpotentials, resulting in an increase in the electrode coefficient of polarization, α. As a consequence, the rate of charge transfer reactions decreases, and the electrochemical performance of the cell might be affected. Thus, the value of the electrode coefficient of polarization is a good index to the quality of SEI which will affect cell SoH degradation rate subsequently.

2. Reduction of Active Electrode Surface Area: The SEI layer can occupy some of the active surface area of the electrode, effectively reducing the available sites for electrochemical reactions. This reduction in the active surface area further affects the current density and can lead to an increase in the electrode coefficient of polarization [1].

3. Stability of SEI Layer: The stability and quality of the SEI layer are critical in determining its impact on the electrode kinetics. A stable and uniform SEI layer can provide a passivating effect, protecting the electrode from further undesirable reactions and electrolyte degradation [3]. However, an unstable or uneven SEI layer can lead to localized variations in charge transfer kinetics and may result in non-uniform electrode behavior. Correspondingly, the value of α will also be unstable if SEI layer is unstable.

4. Li+ Ion Transport: The SEI layer should ideally allow the transport of lithium ions while blocking electrons [3]. The mobility of lithium ions within the SEI layer can affect the overall rate of electrochemical reactions and thus influence the electrode coefficient of polarization [4].

The presence and characteristics of SEI layer are intricately linked to the performance and lifetime of many lithium-ion batteries and other lithium-based electrochemical devices. Proper management and control of the SEI layer formation are essential for maintaining good electrochemical performance and preventing capacity loss and safety issues. In particular, here is how the SEI formation kinetics affect the value of α [5]

1. Early Cycling Effects: During the initial cycles of charging and discharging, the SEI layer forms due to reactions between the electrode material and the electrolyte. At this stage, the SEI layer might not be fully developed or stable. As a result, the charge transfer resistance at the electrode-electrolyte interface may vary during these early cycles [6]. This variability in charge transfer resistance can vary the value of α.

2. Formation of High-Quality SEI: If the SEI layer forms rapidly and uniformly, with good mechanical and ionic conductivity properties, it can serve as a protective barrier between the electrode and the electrolyte. A high-quality SEI layer can mitigate side reactions, prevent further electrolyte decomposition, and reduce polarization effects [7]. This can lead to lower charge transfer resistance and consequently, a lower value of α. It implies that the electrode exhibits more efficient charge transfer kinetics.

3. Formation of Poor-Quality SEI: On the other hand, if the SEI layer forms slowly or is of poor quality, it may have uneven thickness or uneven lithium ion transport properties. This can lead to localized variations in the charge transfer resistance across the electrode surface [8]. As a consequence, the polarization effects become more pronounced, leading to a higher value of α. A poorly formed SEI layer could hinder the rate of electrochemical reactions, reducing the overall efficiency of the cell.

4. Cycling History: The cycling history of the cell, especially during its initial life stages, can affect the formation kinetics of the SEI layer. Variations in temperature, current rates, and cycling protocols can influence the SEI formation process [3] and, in turn, impact the value of α during charge-discharge cycles.

The electrode coefficient of polarization (α) is expected to change with temperature in electrochemical systems. The temperature dependence of α is influenced by several factors related to the underlying physical and chemical processes occurring at the electrode-electrolyte interface. The specific behavior of α with temperature can vary depending on the electrochemical system and the nature of the electrode reactions. Here are some general trends: [9]

1. Activation Energy: The temperature dependence of α is often associated with the activation energy (Ea​) of the charge transfer reactions at the electrode. As temperature increases, the thermal energy available to the reacting species also increases, leading to a higher likelihood of overcoming the activation energy barrier. This can result in faster charge transfer kinetics and a decrease in the value of α [9].

2. Ionic Mobility: Temperature affects the mobility of ions in the electrolyte. Higher temperatures enhance the diffusion of ions to and from the electrode surface, which can lead to improved charge transport and lower polarization effects [10]. Consequently, the electrode coefficient of polarization may decrease with increasing temperature.

3. Lithium-Ion Diffusion: The mobility of lithium ions within the electrode materials and across the SEI layer is temperature-dependent. At lower temperatures, lithium-ion diffusion becomes slower, affecting the overall kinetics of the battery. This can lead to higher polarization and a higher value of α[10].

4. Phase Transition and Solubility: Some electrode materials undergo phase transitions or changes in solubility with temperature variations. These changes can affect the availability of active sites for electrochemical reactions and, consequently, influence the value of α[11].

5. Electrolyte Properties: The properties of the electrolyte, such as its viscosity and conductivity, can be temperature-dependent. A change in these properties can affect mass transport and the overall electrode kinetics, thereby influencing α [10,12].

6. Thermal Expansion: Thermal expansion of electrode materials and the electrolyte can lead to mechanical stress and structural changes, which may affect the electrode-electrolyte interface and alter charge transfer kinetics and α [13]. Nevertheless, it is essential to note that the temperature dependence of α can be complex, and the magnitude and direction of change may vary based on the specific electrochemical system and the nature of the electrode reactions.

The temperature sensitivity of α can be an important factor in understanding the battery's behavior under different temperature conditions, especially during charging and discharging processes. Monitoring how α changes with temperature might provide insights into various aspects of the battery's behavior and can help assess its overall quality. Further work is needed.

**References**

1. Shepherd CM. Design of Primary and Secondary Cells : II. An Equation Describing Battery Discharge. J Electrochem Soc. 1965;112(7):657-64.
2. Choi, Y., Kim, H. and Yoo, J. (2022), Regulating the Polarization of Lithium Metal Anode via Active and Inactive 3D Conductive Mesh Structure. Adv. Energy Sustainability Res., 3: 200065. <https://doi.org/10.1002/aesr.202200065>
3. An SJ, Li J, Daniel C, Mohanty D, Nagpure S, Wood DL. The state of understanding of the lithium-ion-battery graphite solid electrolyte interphase (SEI) and its relationship to formation cycling. Carbon. 2016;105:52-76. <https://doi.org/10.1016/j.carbon.2016.04.008>.
4. Xu N, Shi J, Liu G, Yang X, Zheng J, Zhang Z, Yang Y. Research progress of fluorine-containing electrolyte additives for lithium ion batteries. J Power Sources Adv. 2021;7:100043.
5. E. Peled and S. Menkin 2017 J. Electrochem.Soc. 164 A1703DOI 10.1149/2.1441707jes.
6. Churikov AV. Transfer mechanism in solid-electrolyte layers on lithium: influence of temperature and polarization. Electrochimica Acta. 2001;46(15):2415-26. <https://doi.org/10.1016/S0013-4686(01)00439-X>.
7. Wei L, Jin Z, Lu J, Guo Y, Wang Z, Cao G, Qiu J, Wang A, Wang W. In-situ construction of hybrid artificial SEI with fluorinated siloxane to enable dendrite-free Li metal anodes. Journal of Materiomics. 2023;9(2):318-27. <https://doi.org/10.1016/j.jmat.2022.09.018>.
8. Zhang H, Wang D, Shen C. In-situ EC-AFM and ex-situ XPS characterization to investigate the mechanism of SEI formation in highly concentrated aqueous electrolyte for Li-ion batteries. Appl Surf Sci. 2020;507:145059.
9. Kuratani K, Fukami K, Tsuchiya H, Usui H, Chiku M, Yamazaki S. Electrochemical Polarization Part 1: Fundamentals and Corrosion. Electrochemistry. 2022;90(10):100203. <https://doi.org/10.5796/electrochemistry.2022-66085>.
10. Alipour, M.; Ziebert, C.; Conte, F.V.; Kizilel, R. A Review on Temperature-Dependent Electrochemical Properties, Aging, and Performance of Lithium-Ion Cells. *Batteries* **2020**, *6*, 35. <https://doi.org/10.3390/batteries6030035>
11. Grundy LS, Galluzzo MD, Loo WS, Fong AY, Balsara NP, Takacs CJ. Inaccessible Polarization-Induced Phase Transitions in a Block Copolymer Electrolyte: An Unconventional Mechanism for the Limiting Current. Macromolecules.2022;55(17):7637-49. <https://doi.org/10.1021/acs.macromol.2c00922>
12. Galek P, Slesinski A, Fic K, Menzel J. Peculiar role of the electrolyte viscosity in the electrochemical capacitor performance. J Mater Chem A. 2021;9(13):8644-8654. doi:10.1039/D0TA11230E.
13. Schmider, D.; Bessler, W.G. Thermo-Electro-Mechanical Modeling and Experimental Validation of Thickness Change of a Lithium-Ion Pouch Cell with Blend Positive Electrode. Batteries **2023**, 9, 354. <https://doi.org/10.3390/batteries9070354>
